# Microglia activation in postmortem brains with schizophrenia demonstrates distinct morphological changes between brain regions

**DOI:** 10.1111/bpa.13003

**Published:** 2021-07-23

**Authors:** Ryan Gober, Maryam Ardalan, Seyedeh Marziyeh Jabbari Shiadeh, Linda Duque, Susanna P. Garamszegi, Maureen Ascona, Ayled Barreda, Xiaoyan Sun, Carina Mallard, Regina T. Vontell

**Affiliations:** ^1^ Brain Endowment Bank University of Miami Miami FL USA; ^2^ Centre for Perinatal Medicine and Health Institute of Neuroscience and Physiology Sahlgrenska Academy University of Gothenburg Gothenburg Sweden; ^3^ Department of Clinical Medicine Translational Neuropsychiatry Unit Aarhus University Aarhus Denmark

**Keywords:** microglia, morphology, neuroinflammation, postmortem, schizophrenia, spatial analysis

## Abstract

Schizophrenia (SCZ) is a psychiatric disorder that can include symptoms of disorganized speech and thoughts with uncertain underlying mechanisms possibly linked to over‐activated microglia. In this study, we used brain samples from sixteen donors with SCZ and thirteen control donors to assess the differential activation of microglia by quantifying density and 3D reconstruction of microglia stained with ionized calcium‐binding adaptor molecule‐1 (Iba1). Our samples consisted of sections from the frontal, temporal, and cingulate cortical gray matter, subcortical white matter regions (SCWM), and included the anterior corpus callosum. In the first series of studies, we performed a density analysis followed by a spatial analysis to ascertain the microglial density, distribution, and soma size in SCZ brains. Second, we performed a series of morphological quantification techniques to investigate the arborization patterns of the microglia in SCZ. The results demonstrated an increase in microglia density in the cortical gray matter regions in SCZ cases, while in the SCWM, there was a significant increase in microglia density in the frontal and temporal, but not in the other brain regions of interest (ROIs). Spatial analysis using the “nearest neighbor” demonstrated that there was no effect in “clustering”, but there were shorter distances between microglia seen in the SCZ cases. The morphological measures showed that there was a region‐dependent increase in the microglia soma size in the SCZ cases while the Sholl analysis revealed a significant decrease in the microglia arborization in the SCZ cases across all the ROI’s studied. An in‐depth 3D reconstruction of microglia in Brodmann area 9 cortical region found that there was a significant association between age and reduced microglial arborization in the SCZ cases. This region‐dependent age association can help determine whether longitudinal changes in microglial activation across age are brain region‐dependent, which may point to potential therapeutic targets.

## Graphical abstract

Morphological alterations seen in postmortem brain samples of people with schizophrenia may indicate a shift towards increased activation. The anatomical data shown in this study demonstrates regional changes in microglia.

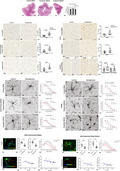

## 1 INTRODUCTION

Schizophrenia (SCZ) is a complex disorder that has a diverse range of phenotypic presentations. Symptoms of SCZ include (1) delusions, (2) hallucinations, (3) disorganized speech, (4) disorganized or catatonic behavior, and (5) flat or blunted affect/negative symptoms. In Diagnostic and Statistical Manual of Mental Disorders‐5 (DSM‐5), 1 of the 5 aforementioned symptoms were required if hallucinations included a running commentary on a person's thoughts and behaviors (1). The psychosis has an onset in adolescence and is significantly debilitating and interferes with day‐to‐day affairs. The neurodevelopmental hypothesis of SCZ proposes that SCZ results from alterations in neuronal connectivity occurring during neuronal development (2). In line with this hypothesis, there has been evidence of increased production of pro‐inflammatory cytokines recognized by over‐activating microglia during fetal gestation (3) and well into adulthood (4, 5). The consequences of over‐stimulated microglia from prolonged exposure to an inflamed or excitotoxic environment during neuronal development may cause over‐pruning of synapses by microglia because they are predisposed to inflammatory mediators (6, 7). It has been suggested that over‐stimulated microglia also play a role during early adulthood, as they have been shown to disturb cortical maturation and affect synaptic pruning during adolescence in populations that are considered at high risk for psychosis (8, 9).

Microglia cells are a heterogeneous cell type derived from primitive myeloid precursors cells (i.e., formed outside of the brain), which give rise to the monocyte‐macrophage‐like lineage (10, 11, 12, 13). Microglia can also come from primitive macrophages that differentiate in the yolk sac early in development (13). Microglia are continuously surveying the central nervous system (CNS), and besides responding to inflammatory insults, these cells support synaptic addition, elimination, and plasticity (14, 15, 16). In cases of SCZ, it is proposed that an increase in monocytes and lymphocytes are recruited from the peripheral blood and exaggerate the CNS immune responses by cooperating with the brain‐derived microglia (4, 17). When considering the differentiaion between residual and paranoid SCZ, using the DSM‐4 criteria, it has been shown that the densities of CD3^+^ and CD20^+^ lymphocytes in the hippocampus were significantly higher in the SCZ groups versus the control groups which indicated diverse immune endophenotypes in SCZ (18).

Historically microglia cells have been classified by their morphology; amoeboidal (i.e., activated), ramified (i.e., quiescent), and intermediate (19). However, recently it has been suggested to classify microglia based on the functional nomenclature; quiescent/surveying, reactive/activated, and phagocytic (20). These morphological changes allow the microglia to acquire diverse and complex phenotypes to participate in the cytotoxic response, immune regulation, and injury resolution. Besides morphological changes, once activated, microglia will upregulate the expression of major histocompatibility complex (MHC) from class I to class II molecules on their cell surface and become more amoeboid and phagocytic (3, 21). Additionally, it has been established that microglia are sensitive to their surrounding microenvironment, meaning that cortical gray matter microglia respond differently to a stimulus than white matter tract bipolar microglia (22, 23). In populations with SCZ, it is thought that a dysregulation of the microglia tends to shift microglia out of the quiescent state into a reactive/activated state, which may exacerberate the periodic schizophrenic episodes (24). Further strengthening the microglial hypothesis is the observation of increased levels of microglial derived cytokines in the CSF of people with SCZ; this also corresponds with increased gene expression levels in microglia (4, 25). Based on these observations, it has been suggested that microglial differences and resulting cytokine expression changes can be used to tease apart the biology underlying each distinct SCZ phenotype (18). Existing studies measuring microglial changes, such as cortical microglia density in the SCZ condition, have shown conflicting results and only offer a limited interpretation (3, 26, 27). One solution is to classify cases with SCZ by correlating gene expression and the levels of pro‐inflammatory cytokines with the state of psychosis (e.g., paranoid SCZ versus residual SCZ) (18) as suggested in DSM‐4. It was shown that populations experiencing active psychosis (paranoid SCZ group) have a dramatic increase in pro‐inflammatory cytokines compared to patients in a non‐acute or quiescent state (residual SCZ group) (18, 28). Patients diagnosed with SCZ have shown increases in circulating pro‐inflammatory cytokines, including interleukin 6 (IL‐6), IL‐8, and IL‐1β, which might be important for driving microglial activation in the disease (27, 28). Complementing this hypothesis, studies using immunohistochemical markers specific to microglial activation (24) and cytokine quantification have found a correlation between structural brain changes in people with SCZ and microglial activation alongside cytokine production (4, 24, 28, 29).

With an increased interest in the microglial component of SCZ, it is vital to include studies that focus on assessing microglial densities in individuals with a long‐term history of SCZ (3, 24, 30), but do not have any other underlying neurodegenerative conditions (e.g., Alzheimer's pathological changes). In‐depth analysis of microglia morphology and spatial distributions will not only complement the findings seen in studies that report an increase of microglial activation alongside structural brain changes (8) but will also shed light on if microglia actively recruit and proliferate during times of active psychosis. Recently, it has been shown that microglia are seen in deeper cortical layers and in adjacent subcortical white matter (SCWM) structures contribute to the structural and functional dysconnectivity of oligodendrocytes in white matter structures (31), which demonstrates a need to analyze the morphological changes in both deeper cortical and SCWM regions. In the current study, we aimed to characterize brain regional microglial changes in patients diagnosed with SCZ for at least 12 years, without any other neuropathological changes (e.g., Alzheimer's neuropathological changes). This study concentrated on the microglia cell densities in postmortem brains of middle‐age donors with SCZ and age‐matched unaffected controls in three brain regions (dorsolateral prefrontal, superior temporal, and anterior cingulate), including both the cortical gray matter and SCWM for each region, as well as the corpus callosum (anterior cingulate). These three cortical gray matter regions have been cited as areas of concern in both *in vivo* PET and postmortem studies (3, 5, 32), but less is known about the SCWM and the adjacent corpus callosum.

We also extended our study to include a detailed morphological analysis of microglia to report activation stages and surveillance behavior in SCZ. We hypothesize that in SCZ, there is a higher density of microglia and a shift towards activated morphologies, which would correspond with the increased cytokine levels seen in cases with SCZ. Finally, we aimed to characterize distribution patterns of microglia using a “nearest neighbor analysis” to better understand the recruitment and proliferative behaviors of microglia in SCZ. An improved understanding of how microglia interact with one another and the surrounding environment will provide new insight into microglia's role in the pathology of SCZ. Enhanced understanding of microglial changes and mechanisms of microglial activation can potentially help to identify novel therapeutics aimed at mitigating aberrant microglial activity in SCZ.

## 2 MATERIALS AND METHODS

### 2.1 Postmortem human brain

Informed consent was acquired for postmortem examination research according to the University of Miami, Institutional Regulatory Board (IRB) guidelines. Research study ethics was obtained from the Human Subjects Research Office, University of Miami, Miami, Florida (IRB ethics number, 19920348; CR00012340) Brain Endowment Bank.

### 2.2 Tissue preparation

Sixteen postmortem brains from donors with SCZ (average age 48 ± 10.2 years; 6 females and 10 males) were used in this study, and 13 age‐matched healthy controls (average age 54 ± 10.7 years; 4 females and 9 males). The diagnosis of SCZ was provided by a psychologist or psychiatrist as noted in the patient charts. Cases with clinical findings of dementia, Alzheimer's changes, or other neurodegenerative diseases were excluded from the study. The primary cause of death in each case was given by the clinician and confirmed by the pathologist. None of the brains showed any significant pathology on gross and microscopic examination. In brief, whole brains were procured on a postmortem interval (PMI) of an average of 24 ± 13 h after the time of death. The left hemisphere was frozen, and the right hemisphere was fixed in 10% formalin (pH 7.0) for 1 month and was sliced, sampled, and embedded in paraffin blocks that were processed on a Leica Tissue‐Tek Processor (Leica Biosystems, Buffalo Grove, IL, USA). Paraffin‐embedded tissue sections were taken from the following regions: the dorsolateral prefrontal cortex Brodmann area 9 (BA9) gray matter and SCWM, the superior temporal cortex (BA22) gray matter and SCWM, and the anterior cingulate cortex (BA32) gray matter and SCWM, with the adjacent anterior corpus callosum and were used for immunohistochemical and histochemical staining.

The paraffin‐embedded tissue blocks were sectioned at 20‐μm thickness with 20 serial sections retrieved using a Leica RM2245 microtome (Leica Microsystems Ltd.). Three sections based on a systematic sampling principle and a section‐sampling fraction of 1/5 (33) were selected from each block for further investigations.

### 2.3 Immunohistochemistry and histochemistry

Standard immunohistochemistry procedures for 20 μm‐thick brain sections have been described previously in (34, 35) with the addition of Methyl Green counterstain being substituted for hematoxylin in the immunohistochemical stain. In brief, after deparaffinization, endogenous peroxidase activity was quenched by placing the slides into 3% hydrogen peroxide (H_2_O_2_) for 10 min. Sections were immersed in preheated 10 mM citric acid (VWR, Radnor, PA, USA), pH 6.0, for 30 min and cooled in cold water. Sections were then blocked in 5% goat serum (Vector Laboratories, Burlingame, CA, USA) for 20 min before being incubated overnight at 4℃ in a solution of rabbit anti‐ionized calcium‐binding adapter protein‐1 (Iba1; 0.1 μg/ml; WAKO Chemicals, Richmond, VA, USA) antibody in PBS. The next day, the sections were exposed to the biotinylated goat‐anti‐rabbit IgG secondary antibody (15 μg/ml; Vector Laboratories) in PBS for 1 h followed by avidin‐biotin complex for 1 h (1:200, ABC; Vector Laboratories). The reactions were visualized with 3,3′‐diaminobenzidine (Millipore‐Sigma, St. Louis, MO, USA) for 10 min. Finally, the sections were dehydrated, cleared in xylene and cover slipped. As negative controls, we performed staining in the absence of the primary antibodies and no specific staining was identified in these preparations (See Data S2, Figure S3).

### 2.4 Histochemistry

The standard tissue paraffin block was sectioned at 20‐μm thickness and the slides were allowed to dry and heated at 60℃ for 30 min. Prior to staining, sections were deparaffinized in three changes of xylene and rehydrated through graded concentrations of ethanol. The histological hematoxylin and eosin (H&E) staining was used to evaluate the general morphology of the brain tissue and the orientation of the brain regions as described previously (36).

### 2.5 Microscopic analyses

#### 2.5.1 Cortical thickness

**FIGURE 1:**
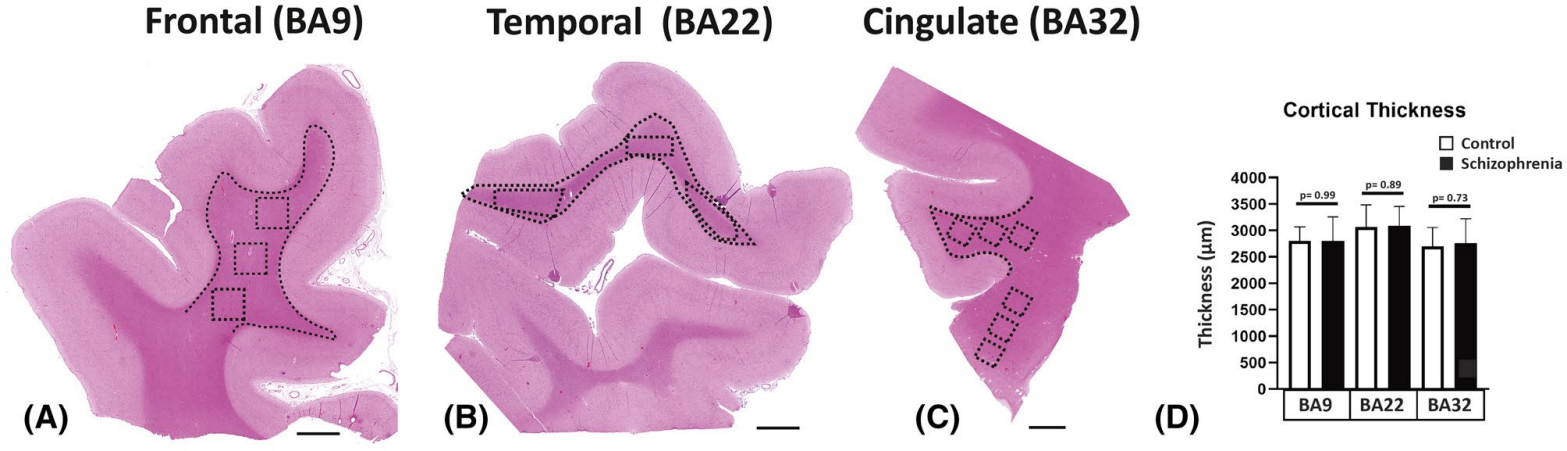
The global view and the strategy for the quantification of the microglia density outlined in the hematoxylin and eosin stain. The regions of interest included the dorsal lateral frontal cortex (BA9; A), the superior temporal cortex (BA22; B), and the anterior cingulate cortex (BA32; C). The areas that were analyzed are represented by dotted lines which show the contours made in each cortical region, and the boxes represent the adjacent subcortical white matter (SCWM) regions and corpus callosum region. Cortical thickness (D) was measured in images A–C. BA, Brodmann area; Scale bar = 3mm

The images of the H&E‐stained sections were obtained using the standard virtual tissue scan (EasyScan, Motic Microscopes) at a 40X magnification. Unbiased measurement of the thickness of the cortex was done using the “Incremental Distances” plugin (Image‐Pro Premier; Media Cybernetics) to measure the distance between the white matter boundary and the edge of the pia matter (i.e., directly above cortical layer 1) using perpendicular lines with a 10 µm step to give an average of 1000 measures, in an average cortical area of 3 mm^2^ per slide. Sampling areas and the strategies for identifying regions of interest were shown using a standard H&E stained section (Figure 1A–C).

### 2.6 Microglia cell density

Unbiased cell densities of Iba1 positive microglia in 1 mm^3^ of tissue were obtained using the extended Depth of Field (EDF) virtual tissue scan, which allowed for a series of Z‐stack images to be transformed into a single image (EasyScan, Motic Microscopes, Schertz, TX, USA). Three contours from each region of interest (ROI) were taken from the cortical gray matter, the adjacent SCWM, and the corpus callosum, which encompassed an average area of 5.4 mm^2^ per region using the Image‐Pro Premier (Media Cybernetics, Warrendale, PA, USA) program.

Three cortical ROIs were determined by the cellular architecture described in (37) and sampled from the postmortem brains: BA9 was sampled from the cortical gray matter and SCWM, BA22 was sampled from the cortical gray matter and SCWM and BA32 was sampled from the cortical gray matter, SCWM and the anterior corpus callosum. For each region, the cortical gray matter scans encompassed cortical layers IV, V, and VI. The SCWM scans were taken in areas adjacent to the cortical ROIs, and the corpus callosum was sampled from the genu inferior to the anterior cingulate.

The cellular densities of Iba1 positive microglia in all the contours were quantified by authors who were blinded to case data. Tissue scans were reviewed (by RV) to ensure that the microglia had met the criteria to avoid duplicate counts (e.g., an area containing a positive nucleus [>10 μm^2^] and connected Iba1 positive processes). In the pilot study, we confirmed that the counting profile described previously counted the correct number of labeled cells and nuclei (using an Image J cell counter).

Estimation of number density was performed by applying the following formula (38):

$$N=\frac{\Sigma Q^{‐}}{V}$$

where *N* is the total number of microglia per volume of brain region; ΣQ^–^ is the number of counted microglia; *V* is the volume of regions of interest per sampling frame.

### 2.7 Microglial spatial analysis

Average nearest neighbor distances (NND) were calculated for each case in a cohort of 19 postmortem brains (n = 9 control, n = 10 SCZ) using the “nearest neighbor distance” plugin on ImageJ (National Institutes of Health) (39, 40). The cohort was chosen based on clear immunohistochemical results and cases with background noise were excluded from this series of studies to ensure consistent thresholding and microglia population representation in the experiment. Individual microglia that fit the criteria (e.g., cell body [>15 µm^2^] and connected Iba1 positive processes) for the analysis were isolated from the sample regions using thresholding adjusted for differences in background intensity (Data S1, Figure S1). Microglial distribution was measured by calculating the R‐value for each case by dividing the average NND value by a hypothetical “random nearest neighbor distance” (41).

### 2.8 Microglia soma size and soma/cell size ratio

The soma size to cell size ratio was calculated using the parent‐child application (Image‐Pro Premier; Media Cybernetics). For each ROI, we defined the measured area (μm^2^) of Iba1 immunoreactivity nucleus, cytoplasm, and processes as the “parent” and the soma area as the “child.” Differentiation between the “parent” and “child” regions was achieved using thresholding and size constraints (Data S2, Table S1) (35). The average “child” area (15 µm^2^) was used to set the threshold minimum and maximum to register how much signal (“child”) was found in each cell body.

### 2.9 Sholl analysis

Sholl analyses to assess microglial morphology in BA9, BA22, and BA32 were performed according to the Sholl Analysis Plugin (Image J, NIH). In BA9, an additional Sholl analysis was performed together with 3D reconstruction of microglia using Filament Tracers algorithm in Imaris software (Version 8.4, Bitplane A.G., Zurich, Switzerland) to construct and trace fine branching details that were not captured on the Motic Microscope. Concentric circles for the analyses began with an initial starting radius of 7‐μm centered on the soma of individual microglia and proceeded outwards in 5‐μm increments. All intersection data from individual microglia in each region were merged into individual profile plots for comparing the overall morphology of control and SCZ microglia as described in Contestabile et al. and Morrison et al. (42, 43).

### 2.10 Image acquisition, 3D reconstruction and morphological analysis of microglia

A total of 30 Iba1 positive microglia in the BA9 (15 cells in cortical and 15 cells SCWM) area were selected in each case for 3D reconstruction and morphological analysis in connection with microglia activity. A systematic set of Z‐stacks of the ROI on three Iba1 stained sections were captured by using the newCAST software (Visiopharm, Hørsholm, Denmark) on a light microscope (Leica DM6000 B, Germany) modified for stereology with a digital camera (Leica DFC 295, Germany), a motorized microscope stage (Ludl MAC 5000, United States) and a 63X oil‐immersed objective lens. Selection criteria for microglia were (1) cell bodies must be in the middle of the section thickness with a clear border, (2) all branches are intact, and (3) branches of the cell should be easily distinguishable from other cells or background staining. A systematic set of Z‐stacks of images was obtained using z‐plane step size of 1‐µm by selecting the middle of section as zero (44). The captured images were analyzed by using Filament Tracers algorithm in Imaris software.

### 2.11 Data analysis

In this study, we used independent t‐test as a parametric test to compare the variables between two groups (SCZ and control) in all experiments (density, distribution, and morphology). Prior to the statistical tests, two assumptions, normal distribution of data and variance homogeneity, were checked by making a Q–Q plot of the data and using Bartlett's test, respectively. Two‐tailed Pearson analysis was used to test the correlation between different morphological parameters of microglia and age. Spatial‐temporal association rules were used to predict the disease (SCZ) based on the length and number of microglia processes as conditions by indicating % of condition support (% of rules that has disease in the condition) (45, 46). Partial Eta square (*η* ^2^) for the effect size of disease on the morphological changes of microglia was calculated by considering observed power using alpha = 0.05 based on the following formula:

$$\eta^{2}=\frac{\mathrm{SSbetween}}{\mathrm{SStotal}}$$

where SS = sum of squares.

Data were presented as mean ± standard deviation (SD); significance was assumed at *p* < 0.05. All statistical tests were two‐tailed. All statistical analyses and generation of plots were performed using GraphPad Prism 8.0 (GraphPad Software, San Diego, CA).

## 3 RESULTS

These experiments were designed to determine the density, distribution, and morphology of microglia in middle‐age, postmortem human brains with and without SCZ disorder. In the control group, the causes of death were complications because of cardiovascular disease (7 donors), respiratory failure (1 donors), accidental deaths (4 donors), and undetermined causes (1 donor). The donors with SCZ had similar complications as the control group, but were diagnosed with SCZ and died from cardiovascular disease (8 donors), respiratory failure (2 donors), accidental deaths (2 donors), or suicide (4 donors). The ages of the control donors were not significantly higher than that of the donors with SCZ (*p* > 0.05), and both groups had similar daily nicotine habits. Additionally, the two groups were not significantly different with respect to the PMI time (*p* > 0.05). Demographics are shown in Table 1.

**TABLE 1:** Summary of the clinical information of the human postmortem cases

Group	Age (years)	Sex	PMI (h)	Ethnicity	Neurolopathological findings	Nicotine use	Cause of death	Manner of death	Approximate (years) diagnosed with SCZ	Neuroleptics
SCZ1	48	F	48.00	Caucasian	Normal brain	N	Myocardial infarction	Natural	30	Unknown
SCZ2	55	F	20.75	Caucasian	Normal brain	H	Blunt force trauma	suicide	35	Unknown
SCZ3	49	M	21.00	Caucasian	focal meningitis	U	Bronchopneumonia	Natural	25	Unknown
SCZ4	40	M	19.00	Caucasian	Normal brain	H	Cardiopulmonary arrest	Natural	23	Unknown
SCZ5	53	F	17.00	Caucasian	Mild anoxic ischemic changes	H	Food asphyxiation	Accidental	30	Unknown
SCZ6	62	F	54.72	African American	Normal brain	H	Cardiopulmonary arrest	Natural	42	Haloperidol
SCZ7	34	F	25.00	Caucasian	Normal brain	L	Accidental	Accidental	16	Unknown
SCZ8	30	F	24.10	African American	Normal brain	M	Gunshot	Suicide	12	Unknown
SCZ9	36	M	24.00	Caucasian	Normal brain	M	Hanging	Suicide	22	Olanzapine
SCZ10	62	M	11.41	Caucasian	Normal brain	M	Acute coronary insufficiency	Natural	40	Clozapine
SCZ11	55	M	13.00	Caucasian	Normal brain	M	Sepsis because of pneumonia	Natural	40	Fluphenazine
SCZ12	56	M	19.00	Caucasian	Normal brain	N	Arteriosclerotic heart disease	Natural	36	Chlorpromazine
SCZ13	42	M	17.00	Caucasian	Normal brain	H	Cardiomegaly	Natural	32	Quetiapine
SCZ14	39	M	18.00	Caucasian	Normal brain	M	Arteriosclerotic heart disease	Natural	20	Quetiapine
SCZ15	59	M	24.00	Caucasian	Normal brain	H	Hanging	Suicide	40	Haloperidol
SCZ16	46	M	46.00	Caucasian	Normal brain	N	Cardiopulmonary arrest	Natural	28	Haloperidol
Mean	48		25.12						29.44	
C1	63	M	20.50	Caucasian	Normal brain	H	Cardiac arrest	Natural		
C2	63	F	17.70	Caucasian	Normal brain	M	Coronary artery disease	Natural		
C3	54	F	17.51	Caucasian	Normal brain	N	Motor vehicle accident	Accidental		
C4	34	M	28.35	Caucasian	Normal brain	N	Drowning	Accidental		
C5	60	M	25.88	Caucasian	Normal brain	L	Electrocution	Accidental		
C6	51	F	15.00	Caucasian	Normal brain	M	Pneumonia, cirrhosis	Natural		
C7	36	M	20.30	Caucasian	Normal brain	M	Undetermined causes	Unknown		
C8	46	M	17.75	Caucasian	Normal brain	L	Gunshot	Accidental		
C9	47	F	20.00	Caucasian	Normal brain	H	Arteriosclerotic heart disease	Natural		
C10	50	M	20.73	Caucasian	Normal brain	M	Acute myocardial infarction	Natural		
C11	66	M	23.28	Caucasian	Normal brain	M	Cardiopulmonary arrest	Natural		
C12	63	M	12.00	Caucasian	Normal brain	M	Coronary artery disease	Natural		
C13	63	M	16.91	Caucasian	Normal brain	N	Arteriosclerotic heart disease	Natural		
Mean	54		19.69							

Abbreviations: C, controls; F, female; H, heavy smoker casual or ≥20 cigarettes/day; h, hours; L, light smoker casual or <1 cigarette/day; M, male; M, moderate smoker ≤19 cigarettes/day; N, nonsmoker; PMI, postmortem interval; SCZ, Schizophenia; U, unknown.

### 3.1 Cortical assessment

Any changes in cellular density in a cortical region could result from shrinkage in the cortical mantle because of neuropil changes. A decrease in cortical volume would be related to a difference in cortical thickness. To address this possibility, the cortical mantle was measured between the gray/SCWM interface and the pial surface. There was no significant difference in the cortical thickness between SCZ and controls in the cortices measured in the BA9, BA22, and BA32 regions (*p* > 0.05; all regions; Figure 1D).

### 3.2 The cell density of Iba1 positive microglia increased in the cortical gray matter in SCZ cases

**FIGURE 2:**
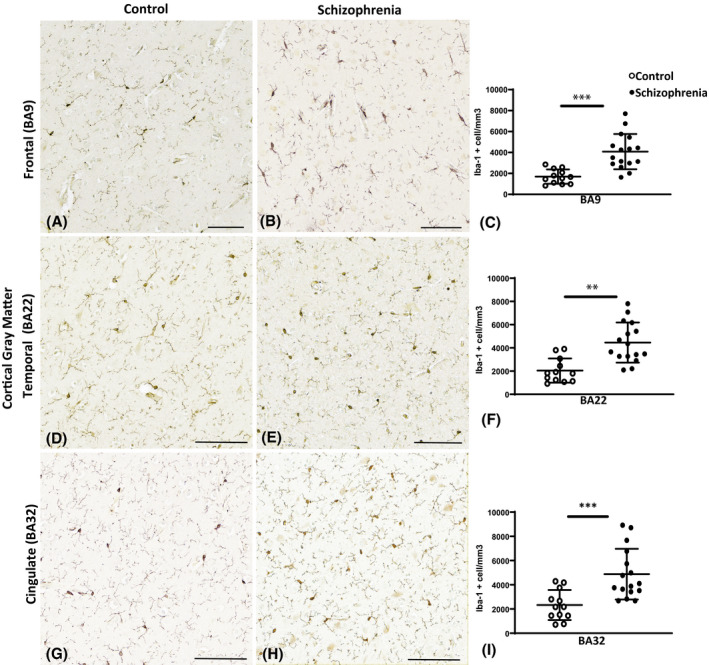
Photomicrographs of the ionized calcium‐binding adaptor molecule 1 (Iba1) immunoreactivity in the control cases (A, D, and G) and in the schizophrenia (SCZ) cases (B, E, and H). Graphs representing the density of microglia per mm^3^ are seen in (C, F, and I). The asterisks indicate the significant differences in the density of microglia counted in the SCZ cases compared to the control in the dorsal lateral frontal cortex (BA9; C), the superior temporal cortex (BA22; F), and the anterior cingulate cortex (BA32; I). BA, Brodmann area; Scale bar = 20 µm. **p < 0.01, ***p < 0.001

In the cortical gray matter, microglial counts showed a significant increase in density of microglia with Iba1^+^ immunoreactivity in the SCZ cases compared to the controls in the dorsolateral prefrontal cortex (BA9; *p* < 0.0001; Figure 2A–C), in the superior temporal cortex (BA22; *p* = 0.002; Figure 2D–F), and in the anterior cingulate cortex (BA32; *p* = 0.0007; Figure 2G–I).

### 3.3 The cell density of Iba1 positive microglia increased in the SCWM in the frontal and temporal lobes in SCZ cases

**FIGURE 3:**
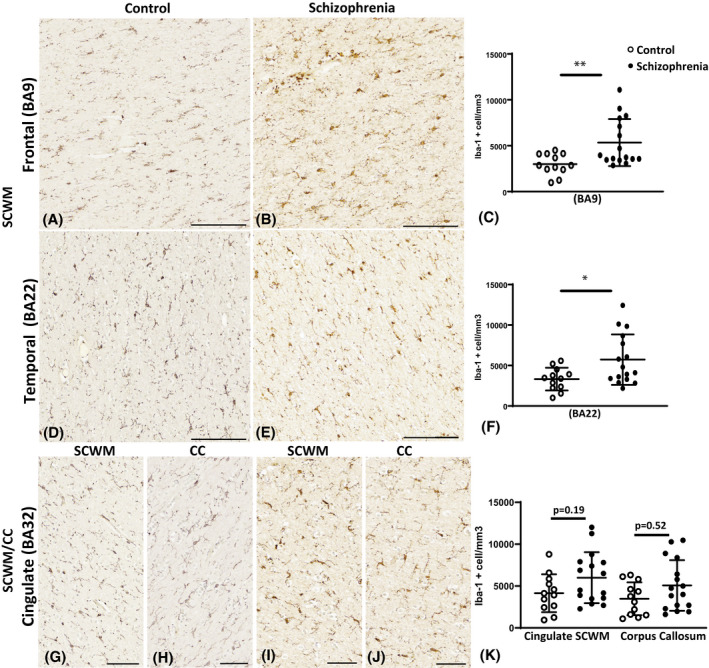
Examples of Iba1 immunoreactivity in the control cases (A, D, G, and H), in the schizophrenia (SCZ) cases (B, E, I, and J), in the subcortical white matter (SCWM) regions, and in the corpus callosum. Graphs representing the density of microglia per mm^3^ are seen in (C, F, and K). The asterisks indicate the significant differences in the density of microglia counted in the SCZ cases compared to the control in the dorsal lateral frontal SCWM (BA9; C), the superior temporal SCWM (BA22; F), and anterior cingulate SCWM and corpus callosum (BA32; K). *p < 0.05, **p < 0.01; BA, Brodmann Area; CC, corpus callosum; Scale bar = 20 µm

A significant increase in the cell density of Iba1 positive microglia in the SCZ cases compared to controls was also found in the SCWM in the prefrontal (BA9; *p* = 0.007; Figure 3A–C) and in the temporal SCWM (BA22; *p* = 0.02; Figure 3D–F). However, microglial cell densities remained unchanged in the cingulate SCWM (BA32; *p* > 0.05; Figure 3G,I,K) and in the corpus callosum adjacent to BA32 (*p* > 0.05; Figure 3H,J,K).

### 3.4 Nearest neighbor measurements demonstrate region specific decreased in microglial proximation in SCZ cases

While increased cell density of microglial in cases of SCZ have been well reported (3), the spatial distributions of microglia in SCZ are less well characterized to date. To quantify spatial changes, the nearest neighbor analysis was performed which measured the (NND) for each microglia (see Data S1; Figure S1 for an overview of the analysis). In BA9, the nearest neighbor analysis produced a significant decrease in the NND distances in both the cortical gray matter and SCWM (*p* = 0.001; *p* = 0.01) in the SCZ cases compared to controls. In BA22, there was no significant change in NND in the cortical gray matter (*p* = 0.530), but there was a significant decrease in the SCWM of the SCZ cases compared to controls (*p* = 0.015). In BA32, there was a significant decrease in the NND in the cortical gray matter (*p* = 0.009) and in the corpus callosum (*p* = 0.04), but not in the SCWM (*p* = 0.062) (Table 2). The data suggest that in cases of SCZ, there are cortical regions where microglia exist in close proximity to one another, which may exacerbate pro‐inflammatory signaling.

**TABLE 2:** Average nearest neighbor measurements and spatial calculations of microglia

Region	NND (µm)				R‐value			
	Control	Schizophrenia	*p* value	*p* value summary	Control	Schizophrenia	*p* value	*p* value summary
BA9	69.5 ± 12.1	50.7 ± 5.4	<0.001	***	1.109 ± 0.021	1.092 ± 0.018	0.654	ns
BA22	62.3 ± 8.7	53 ± 10.7	0.53	ns	1.093 ± 0.013	1.078 ± 0.023	0.454	ns
BA32	56.6 ± 7.78	46.74 ± 6.6	0.009	**	1.080 ± 0.012	1.092 ± 0.014	0.558	ns

Region	NND (µm)				R‐value			
	Control	Schizophrenia	*p* value	*p* value summary	Control	Schizophrenia	*p* value	*p* value summary
BA9 SCWM	49.2 ± 10.1	37.8 ± 3.8	**0.01**	**	1.101 ± 0.015	1.123 ± 0.013	0.953	ns
BA22 SCWM	46.1 ± 5.4	39.4 ± 5.4	**0.015**	*	1.114 ± 0.013	1.081 ± 0.005	0.104	ns
BA32 SCWM	44.2 ± 7.2	38.1 ± 5.8	0.062	ns	1.120 ± 0.007	1.106 ± 0.007	0.943	ns
BA32 CC	34.9 ± 3.0	38.5 ± 3.6	**0.04**	*	1.019 ± 0.102	0.922 ± 0.124	0.155	ns

Note

Data are expressed as mean ± SD. Bold values indicates significant *p* values.

Abbreviations: µm, micron; BA, Brodmann area; CC, corpus callosum; ns, not significant; SCWM, subcortical white matter.

*p < 0.05; **p < 0.01; ***p < 0.001.

### 3.5 Clustering of microglia was not seen in cases of SCZ compared to controls

Because we found differences in the cell densities of microglia in SCZ, our next analysis was to test if the clustering and the distribution patterns differed between SCZ cases and controls in the BA9, BA22, and BA32 regions. The average NND value from each case was used to calculate the “R‐value” (i.e., a calculation to model clustering and distribution patterns), which may be used to interpret how microglia are distributed and if they are being recruited or are proliferating. Calculated R‐values showed that there were no significant differences in clustering and distribution of microglia across all regions in SCZ cases compared to controls (*p* > 0.05; across all regions) (Table 2). This data indicates that in cases of SCZ, proliferation, and recruitment habits of microglia (i.e., clustering) were not altered compared to the controls.

### 3.6 The soma size of microglia is larger in the cortical gray matter in SCZ cases compared to controls

After identifying microglial cell density changes and NNDs in SCZ cases, we set to look for changes in microglial activation by measuring the soma/cell body size ratio. Microglia in control cases were more consistent with a resting morphology (with extensive complex branching and ruffling), whereas in the SCZ cases, microglia consisted of amoeboidal (round to amorphous structures with a variety of short pseudopodia) and intermediate morphologies. The results indicated a significantly larger soma size of microglia in SCZ across the ROI’s (*p* < 0.05) except for the cortical gray matter of BA32 and the corpus callosum (*p* > 0.05). The “parent‐child application” revealed no significant differences in the soma/cell size ratios between the SCZ cases and controls in any of the cortical gray matter regions, the SCWM, and the corpus callosum of any ROI’s (*p* > 0.05). Data summarized in Table 3.

**TABLE 3:** Average cell body to cell size ratios in all regions

Region	Control	Schizophrenia	*p* value	*p* value summary
BA9	0.3349 ± 0.06	0.3969 ± 0.08	0.0331	*
BA22	0.3142 ± 0.09	0.4365 ± 0.11	0.0071	**
BA32	0.3467 ± 0.08	0.3331 ± 0.09	0.6313	ns
BA9 SCWM	0.3347 ± 0.08	0.35 ± 0.11	0.9725	ns
BA22 SCWM	0.3539 ± 0.10	0.4036 ± 0.14	0.341	ns
BA32 SCWM	0.3564 ± 0.09	0.3625 ± 0.11	>0.9999	ns
BA32 CC	0.3208 ± 0.07	0.3964 ± 0.19	0.45	ns

Note

Data are expressed as mean ± SD. Ratios are expressed as average cell body/cell size.

Abbreviations: BA, Brodmann area; CC, corpus callosum; ns, not significant; SCWM, subcortical white matter.

*p < 0.05; **p < 0.01.

### 3.7 A reduction in microglia ramification was observed in the cortex and SCWM in cases with SCZ

Next, we assessed if brains with SCZ showed changes in microglia branching complexity in our ROIs by performing a Sholl analysis, a common technique to measure the arborization of cells by measuring the total numbers of intersections and the maximum process extension length. Microglia have motile processes that extend and retract constantly to monitor and scan the CNS. Microglia in a steady scavenging state, survey the CNS by projecting long filamentous processes that are highly branched and complex which will recede when challenged with a stimulus (47).

**FIGURE 4:**
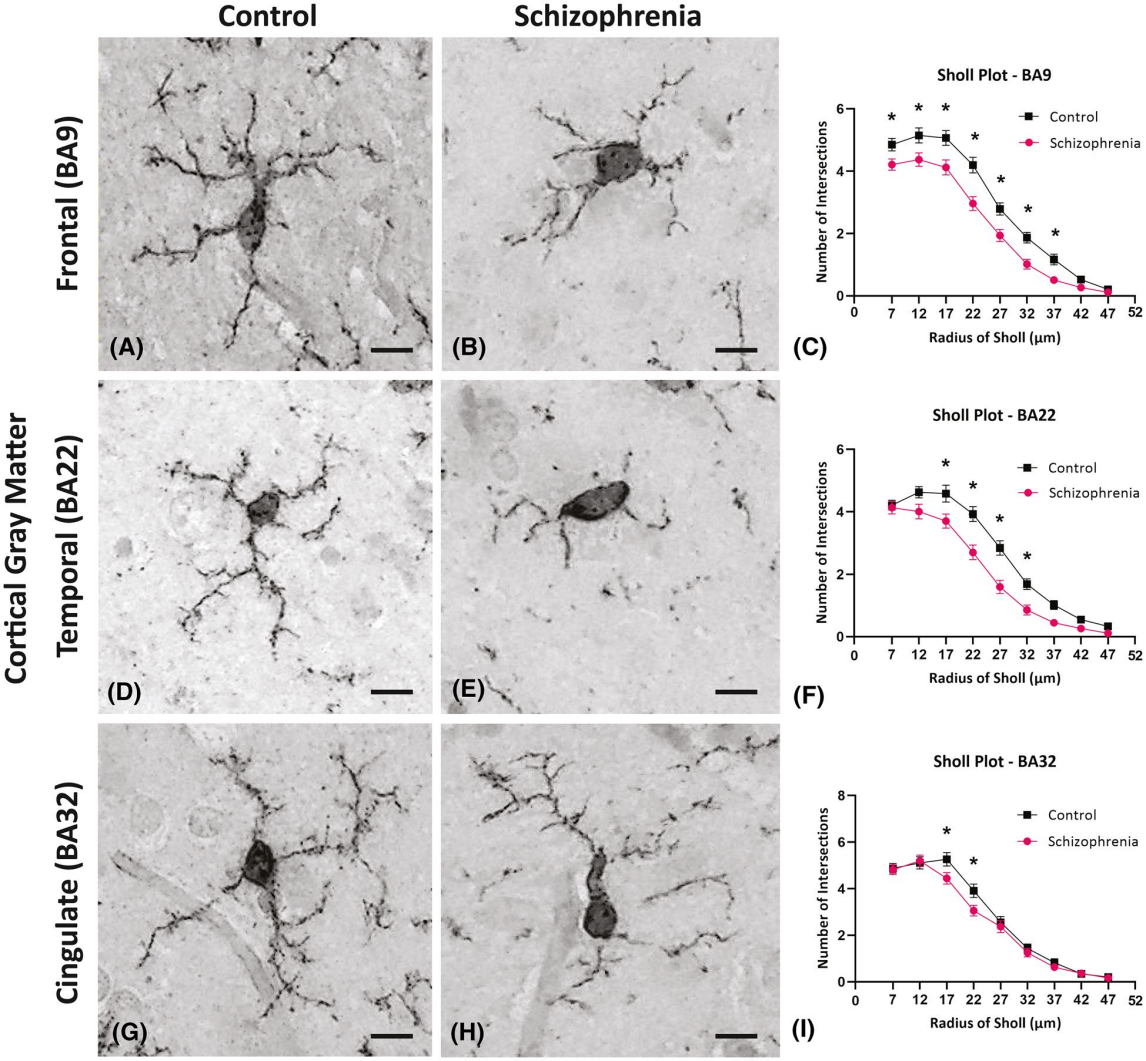
Photomicrographs of cortical Iba1 positive cells used to assess the general morphological differences in microglia in the control cases (A, D, and G) and in the schizophrenia (SCZ) cases (B, E, and H). Inserted above the individual radii in the Sholl plot are asterisks that indicate the significant differences in the microglia branching ramifications in the SCZ cases compared to the control in the frontal cortex (BA9; C) superior temporal cortex (BA22; F) and anterior cingulate cortex (BA32; G). *p < 0.05; BA, Brodmann area; Scale bar = 10 µm

Morphological analysis of microglia in the BA9 region demonstrated that the microglia in this cortical ROI had the most significant overall reduction in the total number of intersections (*p* < 0.001) with a significant reduction of the number of intersections at each radius between 7 and 37 µm (*p* < 0.05) from the cell soma in SCZ microglia compared to the other cortical regions (Figure 4A–C). The BA9 region was thus chosen for a more detailed, 3D reconstruction assay to capture detailed branching changes in relation to age (results in the subsequent section).

Microglial morphology changes were also seen in both the cortical gray matter of BA22 and BA32 in the SCZ cases compared to controls. In the BA22 of SCZ cases, we identified significant reductions in the branching of microglia, as shown by the reduced number of intersections between 17 and 32 µm from the cell soma in SCZ microglia (*p* < 0.05; Figure 4D–F) as well as a lower total number of intersections across all radii (*p* < 0.001). In the same region, we found a decrease in the maximum extension length (*p* < 0.001) of the microglial processes in SCZ cases, indicating a decrease in the overall arborization area and scavenging behavior. In the BA32, cortical gray matter of SCZ cases, microglia exhibit more subtle morphology changes compared to controls (Figure 4G,H) with decreases in the number of branching intersections between 17 and 22 µm from the cell soma (*p < 0.05; Figure 4I). However, there were no significant differences in the total number of intersections (*p* = 0.313) or in the maximum process extension lengths of microglia in this ROI (*p* = 0.097) (Table 4) suggesting less drastic changes in scavenging activity in the BA32 region in SCZ.

**TABLE 4:** Sholl analysis summary

Region	Total number of intersections				Maximum process extension length (µm)			
	Control	Schizophrenia	*p* value	*p* value summary	Control	Schizophrenia	*p* value	*p* value summary
BA9	118.4 ± 47.83	88.55 ± 45.07	**<0.001**	***	37.81 ± 9.62	32.18 ± 8.935	**<0.001**	***
BA22	110.5 ± 46.56	84.06 ± 54.11	**<0.001**	***	38.12 ± 11.30	30.78 ± 9.52	**<0.001**	***
BA32	111.3 ± 51.11	95.40 ± 58.16	0.313	ns	34.80 ± 9.35	31.58 ± 10.58	0.097	ns
BA9 SCWM	60.86 ± 33.69	46.62 ± 27.11	**<0.001**	***	25.36 ± 9.09	23.45 ± 7.44	0.093	ns
BA22 SCWM	51.56 ± 32.34	43.79 ± 41.37	0.129	ns	23.20 ± 7.75	21.11 ± 9.17	0.074	ns
BA32 SCWM	44.84 ± 30.91	27.06 ± 18.29	**<0.001**	***	22.62 ± 9.14	17.64 ± 5.73	**<0.001**	***
BA32 CC	78.91 ± 43.1	71.2 ± 36.16	0.403	ns	32.08 ± 12.38	29.52 ± 8.41	0.398	ns

Note

Data are expressed as mean ± SD. Bold values indicates significant *p* values.

Abbreviations: µm, micron; BA, Brodmann area; CC, corpus callosum; ns, not significant; SCWM, subcortical white matter.

***p < 0.001.

**FIGURE 5:**
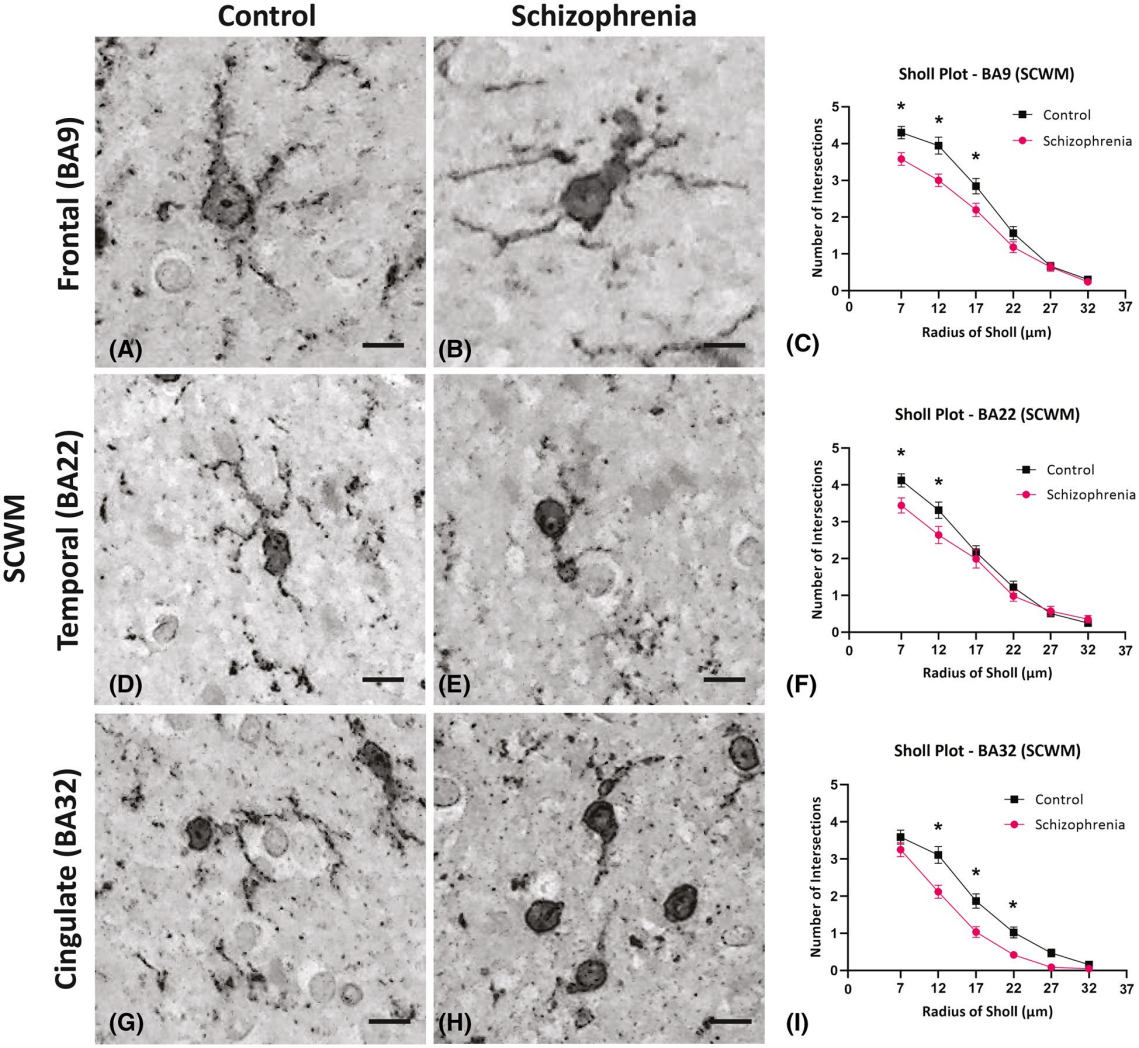
High‐resolution images of Iba1 immunohistochemistry from the subcortical white matter (SCWM) were used to assess the general morphological differences in microglia in the control cases (A, D, and G) and in the schizophrenia (SCZ) cases (B, E, and H). In the Sholl plot, the asterisks indicate the significant differences seen in the microglia branching ramification at the individual radii in the SCZ cases compared to the control cases in the frontal SCWM (BA9; C) superior temporal SCWM (BA22; F) and anterior cingulate SCWM (BA32; G). *p < 0.05; BA=Brodmann Area; Scale bar = 10 µm (A, B); Scale bar = 5 µm (C, D, F, G)

In the SCWM of BA9, there was a significant reduction of the overall number of intersections in SCZ brains compared to control brains (*p* < 0.001) with a reduced number of intersections at each radius between 7 and 17 µm (*p* < 0.05) from the cell soma in SCZ microglia (Figure 5A–C). Microglial morphology changes in the SCWM of SCZ cases were also observed for both BA22 and BA32 regions. In the SCWM of the BA22 region of SCZ cases, there was a decrease in the number of branching intersections 7–12 µm from the cell soma (*p* < 0.05; Figure 5D–F), but no significant changes in the total number of intersections nor the maximum process extension lengths (*p* = 0.129; *p* = 0.75). Conversely, in the SCWM of BA32, we found a more pronounced decrease in the number of intersections that ranged from 12 to 22 µm from the cell soma (*p* < 0.05; Figure 5G–I) and a decrease in both the total number of intersections and the maximum process extension lengths in the SCZ cases compared to the controls (*p* < 0.001; *p* < 0.001). In the anterior corpus callosum, there were no significant changes in the number branches in the anterior corpus callosum 7–27 µm from the cell soma (*p* > 0.05) and no significant changes in the total number of intersections or in the maximum process extension measures (*p* > 0.05) (Table 4). The results from this Sholl analysis showed a region‐dependent decrease in branching and process extension length in SCZ cases, leading to overall decreases in microglial arborization in the SCZ cases. This data suggests that in certain brain regions, the microglia in the SCZ cases tend to have receded processes around the enlarged cell soma, which may be indicative of a more activated phenotype.

### 3.8 Morphological changes of microglia in the frontal cortex (BA9) (gray and SCWM) in cases with SCZ

While BA22 and BA32 have been implicated to be involved in SCZ, recent advances in the understanding of the pathophysiology of SCZ suggest that BA9 is one brain region that is highly impaired in cases with SCZ (25, 48, 49). In addition, ICAM‐1 (Intercellular Adhesion Molecule 1) expression has been found to be correlated with the expression of the macrophage marker CD163, and CD163‐positive macrophages associated with neurons in the frontal cortex of SCZ cases with high levels of inflammation (50). However, how the frontal lobe is affected in schizophrenia especially in connection with neuroinflammation remains unclear. Accordingly, we performed a separate, in‐depth analysis of the 3D morphology of Iba1 immunopositive microglia in BA9 to look for microglial changes that could be linked to higher levels of inflammation in the frontal cortex of SCZ cases.

### 3.9 Total length of microglia processes

**FIGURE 6:**
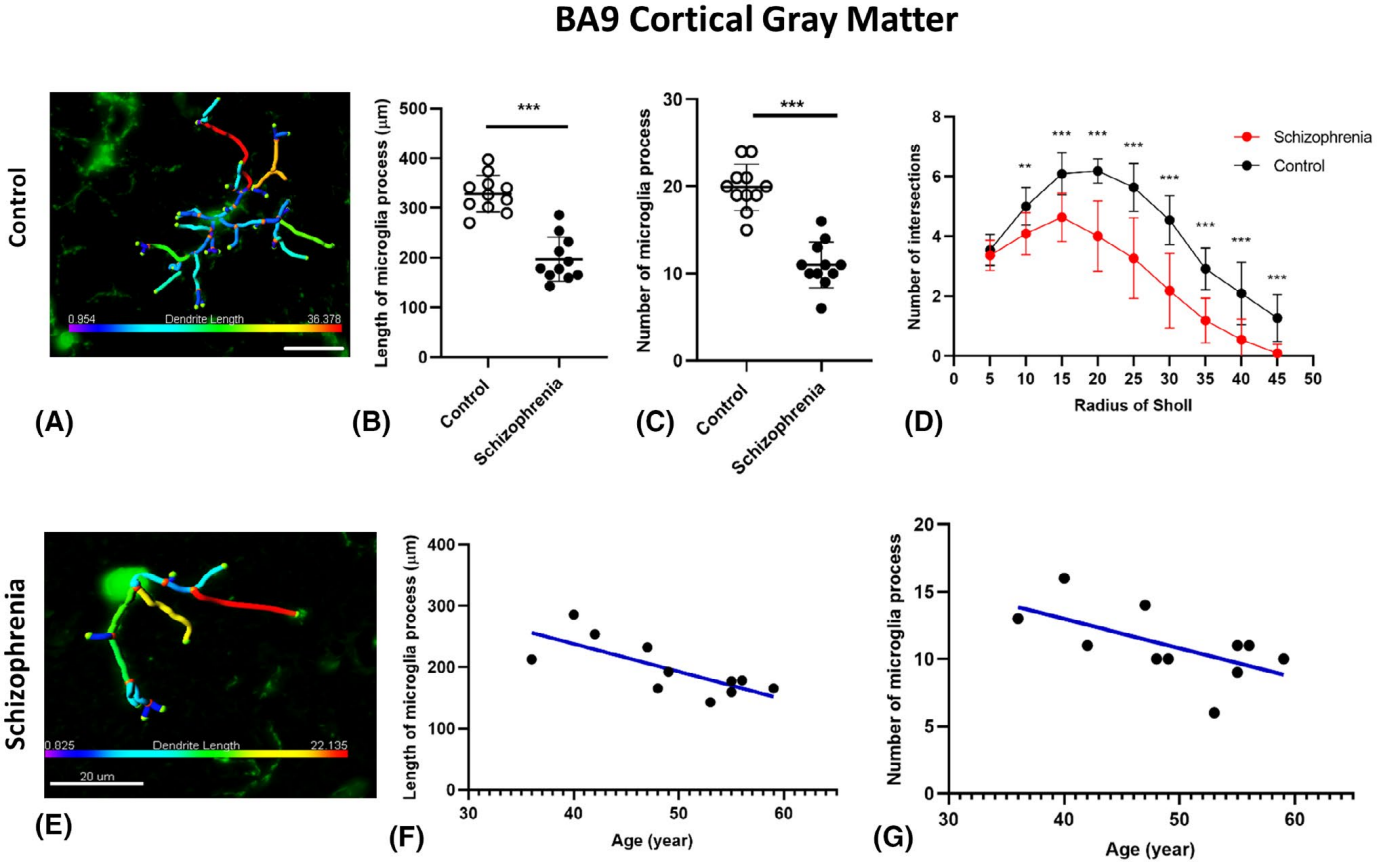
Examples of 3D reconstructed microglia in cortical gray matter in control brain (A) and SCZ brain (E). Microglia morphology indicated less ramification by having a shorter process lengths and smaller numbers of processes in cortical gray matter (B, C) in SCZ group compared with controls. Branching pattern alterations of microglia in cortical gray matter indicated the number of branching intersections was significantly lower in SCZ cases versus the control group (D) at various distances away from the cell soma. Age‐association testing demonstrated a significant negative correlation between ramification of microglia and age of SCZ cases (F, G), **p < 0.01, ***p < 0.001

**FIGURE 7:**
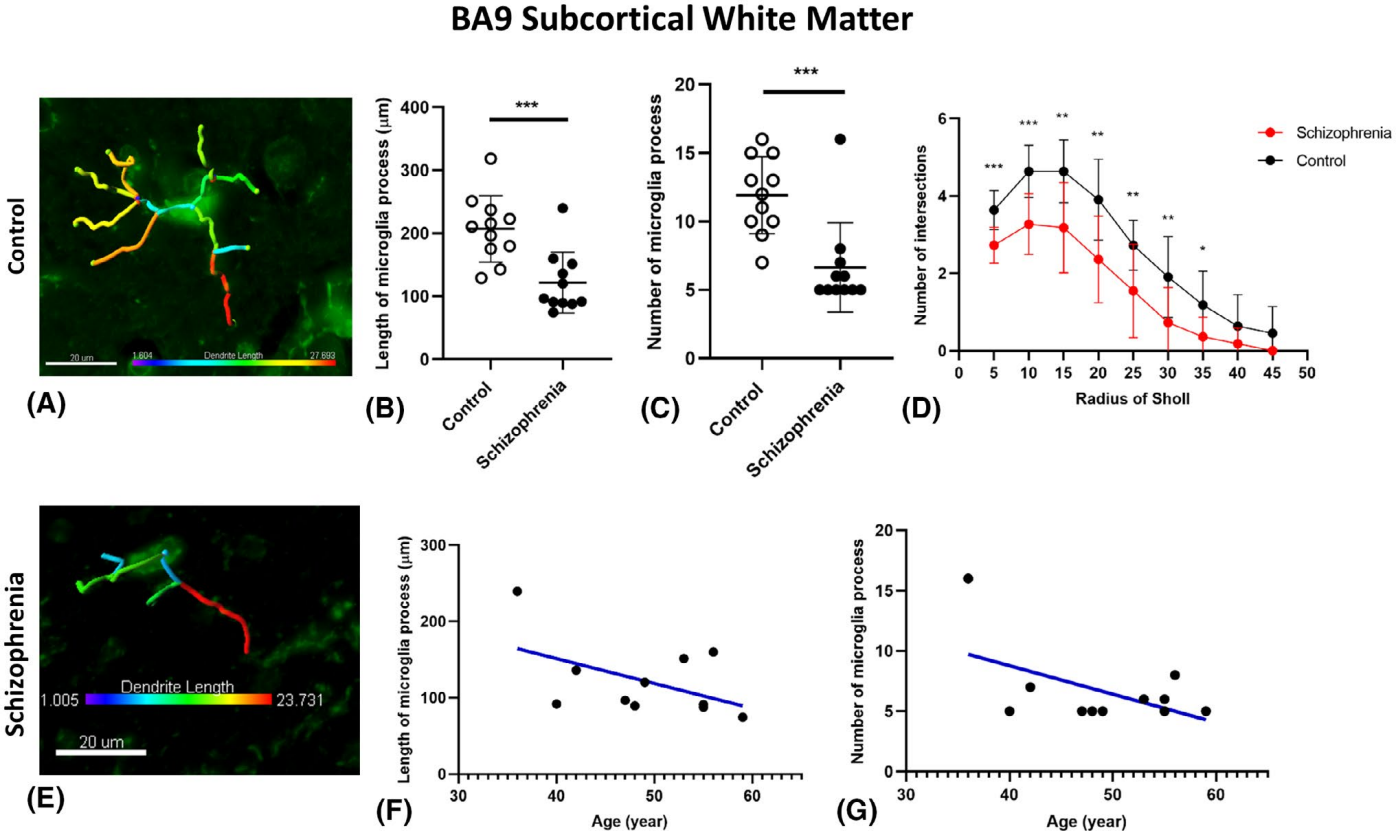
Examples of 3D reconstructed microglia in SCWM in control brain (A) and SCZ brain (E). Microglia morphology indicated less ramification by having shorter and smaller number of processes in SCWM in the SCZ group compared with controls (B, C). Branching pattern alterations of microglia in SCWM indicated the number of branching intersections at various distances away from the cell soma was significantly lower in SCZ cases versus the control group (D). Age‐associated testing demonstrated found no significant correlation between ramification of microglia in SCWM and age of SCZ cases (F, G), *p < 0.05, **p < 0.01, ***p < 0.001

Our results showed that the total length of microglia processes in the cortical gray matter (Figure 6A,B,E) and in the SCWM (Figure 7A,B,E) of SCZ cases was significantly shorter than the control group (*p* = 0.000, *p* = 0.001). Partial Eta square (*η* ^2^) for the effect of disease on the total length of microglia in cortical gray matter and SCWM were 0.74 and 0.44 with the observed power of 100% and 96%, respectively, which indicated a large effect of disease on the length of microglia processes.

Interestingly, our results indicated a significant negative correlation between the total length of microglia processes in the cortical gray matter and the age of the SCZ cases (*r* = −0.75; *p* = 0.008), which suggests that in the younger SCZ cases, the microglia processes were longer and more ramified (less active) than in older SCZ cases (Figure 6F). This correlation was not significant in the control group (Data S2; Figure S2A). Moreover, in both groups no significant correlation between the total length of microglia processes in the SCWM and the age of the cases was observed (*p* > 0.05; Figure 7F). By performing spatiotemporal analysis, we found that in BA9 in SCZ cases, the length of microglia branches ≤193.75 in the cortical gray matter and ≤123.26 in the SCWM can be used as a prediction for the SCZ with the condition support (31.83%) and confidence of 100%

### 3.10 Total number of microglia processes

Microglia in the cortical gray matter and SCWM showed a significantly lower number of processes in the SCZ cases in comparison with the control group (*p* < 0.001, *p* = 0.001; Figures 6C and 7C). Partial Eta square (*η* ^2^) for the effect of disease on the number of microglia processes in cortical gray matter and SCWM were 0.97 and 0.78 with the observed power of 100% which indicated a large effect of disease on the number of microglia processes. In the SCZ brains, there was a significant negative correlation between the number of microglia processes in the cortical gray matter and the age of SCZ cases (*r* = −0.60; *p* = 0.047; Figure 6G), while this correlation was not significant in the SCWM (*r* = −0.53; *p* = 0.092; Figure 7G). Moreover, no significant correlation in the control brains was observed (Data S2, Figure S2B).

By performing spatiotemporal analysis, we found that in the SCZ BA9, in the cortical gray matter 10≤ the number of microglia branches <13 with the condition support (31.82%) and confidence (100%) for SCZ condition can be used as a prediction for the SCZ.

### 3.11 The complexity of microglia processes (Sholl analysis)

In this study, 3D reconstruction and subsequent Sholl analysis revealed that the branching patterns of microglia in cortical gray matter were as significantly different between SCZ and control brains by showing the number of processes intersections at 10–45 μm away from the cell soma (with a radial distance from the center of the microglia soma in 5 µm) were significantly lower in the cortical gray matter of SCZ brains versus control brains (*p* < 0.01; Figure 6D).

In the SCWM, the branching pattern of microglia showed significantly lower numbers of branching intersections at 5–30 μm away from the cell soma (*p* < 0.01; Figure 7D).

## 4 DISCUSSION

In this study, we have quantified microglia using Iba1 expression allowing for cell density and morphological analysis in different cortical, SCWM, and corpus callosum regions in the human postmortem brain with and without SCZ. To the best of our knowledge, this is the first time that an in‐depth analysis of microglia number density, distribution, and morphology has been demonstrated using multiple cortical, SCWM, and callosal regions in postmortem cases with SCZ.

Our data suggest that in cases with SCZ, microglia density is increased in the cortical gray matter of frontal temporal regions and cingulate regions, whereas, microglia cell density was only increased in the frontal and temporal SCWM, but not in the cingulate nor in the corpus callosum. Radewicz et al., using anti‐HLA‐DR antibody, found that in postmortem SCZ donors, there was an increase in microglia numbers in the frontal and temporal cortical regions, but not in the anterior cingulate (3). Antibodies raised against the HLA‐DR antigen, detect MHC class II cell surface receptors (51), which suggests that there may be more activated microglia in the frontal and temporal lobes in cases with SCZ. Our investigation not only substantiates these findings described by Radewicz et al., but also identifies that in SCZ, microglia populations are increased in other cortical regions compared to age‐match controls (3).

In this study, we found that there is an increase in microglia densities in SCWM of the dorsolateral prefrontal and in the superior temporal regions. Interestingly enough, the increase in microglia cell density seen in SCWM regions was found in areas adjacent to cortical regions that had higher microglial density than controls. Increases in microglia densities seen in the white matter tracts have recently gained an interest in studies that range from neonates to aging populations (23, 52). Microglia are required for the maintenance of oligodendrocytes for myelin development in neonates and remyelination in adults (23, 53). An increase in microglia or activation of microglia has been implicated in white matter injuries and in defects in myelination (23). In cases of SCZ, Uranova et al., found that in the prefrontal white matter region, microglia activation contributed to dystrophic changes of oligodendrocytes (31). Furthermore, other studies show that in cases of SCZ, oligodendrocytes have lysed cytoplasm and form vacuoles in the surrounding tissue when they are found in close proximity to amoeboid microglia in cortical gray matter (54). These studies suggest that both cortical and WM activated microglia contribute to an inability to modulate normal brain function, which is exaggerated in cases of SCZ.

Our distribution analyses, which showed shorter average NNDs in the SCZ cases, confirm the BA9 and BA32 density data, as one could infer that the tighter packing of microglia within a given area, should yield a higher density of cells. However, in the BA22 cortex, there was no significant change in the NND’s between SCZ and control cases, although we did find that the microglia packed tighter together in the SCWM of BA22. While this does not explain the abnormality in average NNDs recorded in BA22 compared to microglial cell densities in these regions, it is worth noting that NND measurements may not be sensitive enough to reflect more subtle differences as we had to choose microglia with larger cell bodies to correctly ascertain the NND distance of heterogeneous microglia. Of interest, in the ROIs we did not find a significant change in clustering, suggesting there were no spatial changes of microglia in SCZ compared to control cases. Studies with a focus on microglia densities have consistently shown that in the frontal cortex, there is an increased number of microglia and have indicated that the microglia were potentially showing some degree of activation (i.e., amoeboid morphology) (32, 55). In our data, we did not find microglia clustering in SCZ cases, suggesting microglia proliferation and recruiting may not be occurring. De Picker et al. noted that in SCZ, microglia are central to the abnormal immune response via either remodeling of the extracellular spaces or by the elimination of synaptic plasticity (56). Additionally, other studies have found that it is the placement of the microglia in proximity to the neuron or the oligodendrocyte that may be the key to the dysfunction seen in SCZ (31, 54).

Similar to previous studies, we saw a significant increase in soma size in the BA9 and BA22 cortical regions (24, 30), suggesting a different state of activation of microglia in cases with SCZ patients compared with controls. Despite the variability in density findings of several studies, there is a strong evidence of microglia dysfunction or activation (i.e., cell bodies are more defined, and their processes were less fine) in SCZ (3, 15, 31, 54, 56).

In addition, the loss of ramified microglia identified in the SCZ group across all ROIs, by Sholl analysis in our study and noted in other studies, may indicate interference with neuronal plasticity (54) or excessive synaptic pruning (57). This may underly the alterations in the neuroinflammatory response and results in disorganized thought processes and gray matter atrophy seen in SCZ (57, 58) and other psychiatric diseases, such as depression and autism (59). Indeed, the microglia pathology that we found in this study is not specific to SCZ, rather is involved in the pathophysiological changes of several neurodevelopmental diseases, such as Autism spectrum disorder (ASD) (60, 61). For example, in ASD, it is shown that there is a significant positive correlation between age and primed microglia density in both gray and white matter in the disorder, but not in control conditions.

In SCZ, there are many forms of disease presentation and varying immune and inflammatory states in individual patients suggesting more complex pathophysiological processes may be at play in the disorder. For example, in first‐episode psychosis patients, it has been shown that there is a diverse change in circulating monocyte, lymphocyte, and neutrophil levels which are found to be related to the disease acuity, severity, and the inflammatory state in individual patients. This suggests that such variables may be important in relation to microglial changes (62).

To compound complexity and range of immune changes is the suggestion that there are disruptions of the blood brain barrier (BBB) in SCZ which could lead to differential levels of cytokine and immune cell infiltration in individual patients and further activate microglia (63).

The limitations of postmortem investigations are that it is not possible to exclude the contributions made by other comorbid clinical factors, such as substance abuse, hypertension, or cardiopulmonary complications. We cannot exclude the possibility that the increase in microglia densities seen in the SCZ group may be exacerbated by other clinical conditions. Nevertheless, our data suggest that the changes in density and morphology of the microglia were identified in the SCZ cases when compared to controls with similar medical conditions (e.g., Coronary Artery Disease, Cardiopulmonary Arrest, and smoking history). Unlike other investigations that use fMRI, the small sample size generally used in postmortem studies may limit the possibilities to investigate the contributory role of other clinical conditions. However, the tissue acquired for the current series of investigations, provided the ability to study subtle alterations, in microglial morphology. Importantly, in the current study, we did measure Partial Eta square (*η* ^2^) for the effect of disease on the morphological parameters of microglia to emphases the size of the difference between two groups (SCZ and control) rather than confounding this with sample size.

The current study demonstrates clear microglial changes in several brain regions in SCZ suggesting a microglial component to the pathophysiology of the disorder. Apart from the cortex, the hippocampus is one of the main brain regions which is involved in the pathophysiology of schizophrenia (64). Interestingly, it is shown that microglial activation in different clinical subtypes of schizophrenia is distinct; HLA‐DR+ microglia were increased in the posterior hippocampus of individuals with paranoid schizophrenia relative to those with residual schizophrenia. However, patients with residual schizophrenia had a higher density of CD3+ and CD20+ lymphocytes in the posterior hippocampus (65). Apart from clinical studies, rodent studies showed an increased number of microglial cells together with a reduction in microglia arborization, which suggests microglia activation in the hippocampus in the embryonic rodent following polyriboinosinic‐polyribocytidylic acid (Poly I:C, a viral mimic) exposure (66, 67).

Future studies should aim to document microglial changes in other brain regions implicated in SCZ, such as the hippocampus and the thalamus, to provide a more comprehensive overview of microglial changes across different brain regions.

### 4.1 Conclusion

The present study indicates changes in microglia cell densities and morphologies in cases of SCZ across different cortical regions associated with SCZ (BA9, BA22, and BA32) (see Table 5 for a brief review of the data). We speculate that these morphological alterations may indicate a shift towards increased activation, possibly resulting in functional deficits and playing a role in the disorganized thought processes seen in people with schizophrenia. The anatomical and detailed morphological information gathered from our study provides a solid foundation for future studies of microglia activation in SCZ. The variability seen in the different brain regions in the current study and in several findings indicates that there may also be a genetic or environmental component to microglial dysfunction seen in SCZ. Finally, we report age‐dependent changes in microglia activation in SCZ, which suggests that there may be an age‐related process unique to a person with SCZ.

**TABLE 5:** Summary of microglia changes by region

Region—Tissue type	Microglia density	Microglia nearest neighbor distances	Microglia clustering	Microglia morphology changes		
				Soma size	Overall branching	Extension lengths
BA9—Cortical gray matter	↑	↓	nc	↑	↓	↓
BA22—Cortical gray matter	↑	nc	nc	↑	↓	↓
BA32—Cortical gray matter	↑	↓	nc	nc	↓	nc
BA9—SCWM	↑	↓	nc	↑	↓	nc
BA22—SCWM	↑	↓	nc	↑	↓	nc
BA32—SCWM	nc	nc	nc	↑	↓	↓
BA32—CC	nc	↓	nc	nc	nc	nc

Note

Arrows indicating the increase (up arrow) and decrease (down arrow) of microglia cell densities, distances to the nearest neighbor, clustering pattern, and morphology. nc = no changes in the measurement between schizophrenia (SCZ) and control in the cortical gray matter, subcortical white matter (SCWM), and corpus callosum (CC) regions.

## CONFLICT OF INTEREST

The authors have no duality or conflicts of interest to declare.

## AUTHOR CONTRIBUTIONS

The series of experiments were designed by Regina T. Vontell and Ryan Gober, who completed the cell density, cell body to cell soma ration, and NND measures. The tissue sectioning and immunohistochemistry were completed by Regina T. Vontell and Maureen Ascona. The cohorts for the schizophrenia and control groups were chosen by Linda Duque, and Ayled Barreda. The quality of the tissue was checked by Susanna Garamszegi and the clinical assessments were reviewed by Xiaoyan Sun. The 3‐D analysis, age regression, and Sholl analyses were completed by Maryam Ardalan, Seyedeh Marziyeh Jabbari Shiadeh, and Carina Mallard. All authors assisted in the writing, reviewing, and editing of the manuscript prior to submission.

## ETHICS APPROVAL

Research study ethics was obtained from the Human Subjects Research Office, University of Miami, Miami, Florida (IRB ethics number, 19920348; CR00012340) Brain Endowment Bank.

## Supporting information


**Figure S1** An overview of the computation of the nearest neighbor distances (NNDs) and calculation of a population distribution (R‐value). Examples of microglia captured are seen in (A–C, control; D–F, schizophrenia) where individual microglia were converted to binary (B, E). The Nearest Neighbor Plugin was used to measure the distance of each microglia to its neighboring microglia; allowing the average NND to be calculated (C, F) in each region of interest (ROI). The R‐value equation to calculate the microglial distribution for each ROI as a ratio of the average NND over a hypothetical “random” nearest neighbor value (NND‐r) (G). The R‐value is then used to model whether microglia are distributed in a cluster forma‐tion (R < 1), randomly formation (R = 1) or in a dispersed formation (R > 1; H). [Choe et al. (40); Thumbi et al. (41)]

Supplementary Material
**Figure S2** The correlation between the ramification of microglia and age was not identified in control cases in either the cortical gray matter (A) or the subcortical white matter (B) of BA 9
**Figure S3** A photomicrograph of a negative control from the BA 22 region (A) with higher magnification examples seen from the cortical gray matter (B) and the subcortical white matter region (C)
**Table S1** A review of the measure contraints used in the applications to estimate cell density and morphological changes

## Data Availability

The data that support the findings of this study are available on request from the corresponding author.
