# Supplementary material for: Microglia activation in postmortem brains with schizophrenia demonstrates distinct morphological changes between brain regions
Source: Brain Pathol. 2021 Jul 23;32(1):e13003. doi: 10.1111/bpa.13003 (PMC8713533; doi:10.1111/bpa.13003)
Supplement: Supplementary file 1 — Figure S1 An overview of the computation of the nearest neighbor distances (NNDs) and calculation of a population distribution (R‐value). Examples of microglia captured are seen in (A–C, control; D–F, schizophrenia) where individual microglia were converted to binary (B, E). The Nearest Neighbor Plugin was used to measure the distance of each microglia to its neighboring microglia; allowing the average NND to be calculated (C, F) in each region of interest (ROI). The R‐value equation to calculate the microglial distribution for each ROI as a ratio of the average NND over a hypothetical “random” nearest neighbor value (NND‐r) (G). The R‐value is then used to model whether microglia are distributed in a cluster forma‐tion (R < 1), randomly formation (R = 1) or in a dispersed formation (R > 1; H). [Choe et al. (40); Thumbi et al. (41)] [file BPA-32-e13003-s001.pdf]

Supplementary Data 1

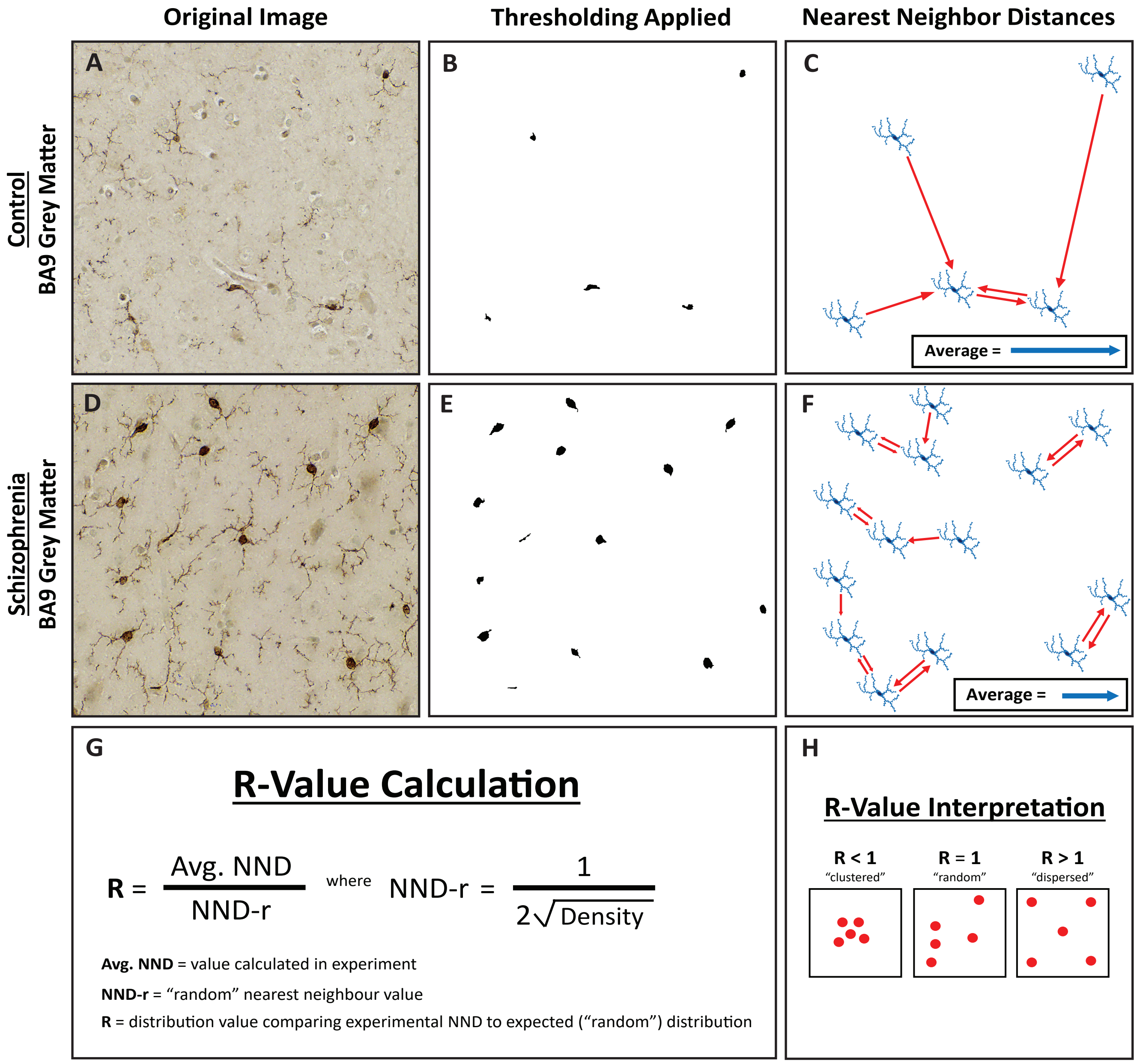

Supplementary Figure 1. An overview of the computation of the nearest neighbor distances (NNDs) and calculation of a population distribution (R-Value). Examples of microglia captured are seen in (A-C, control; D-F, schizophrenia) where individual microglia were converted to binary (B, E). The Nearest Neighbor Plugin was used to measure the distance of each microglia to its neighboring microglia; allowing the average NND to be calculated (C, F) in each region of interest (ROI). The R-Value equation to calculate the microglial distribution for each ROI as a ratio of the average NND over a hypothetical "random" nearest neighbour value (NND-r) (G). The R-Value is then used to model whether microglia are distributed in a cluster formation (R<1), randomly formation (R=1) or in a dispersed formation (R>1; H). (Choe *et al.*, 2016; Thumbi *et al.*, 2010)
