# Supplementary material for: Microglia activation in postmortem brains with schizophrenia demonstrates distinct morphological changes between brain regions
Source: Brain Pathol. 2021 Jul 23;32(1):e13003. doi: 10.1111/bpa.13003 (PMC8713533; doi:10.1111/bpa.13003)
Supplement: Supplementary file 2 — Supplementary Material Figure S2 The correlation between the ramification of microglia and age was not identified in control cases in either the cortical gray matter (A) or the subcortical white matter (B) of BA 9 Figure S3 A photomicrograph of a negative control from the BA 22 region (A) with higher magnification examples seen from the cortical gray matter (B) and the subcortical white matter region (C) Table S1 A review of the measure contraints used in the applications to estimate cell density and morphological changes [file BPA-32-e13003-s002.pdf]

Supplementary Data 2

Measure Constraints Table 1

Analysis	Microglia Size (µm²) Contraints	Thresholding Contraints (Intensity)	Additional Background Adjustments	Analysis Platform
Microglial Cell Density	10- 500	(15 - 165)	8 Pixel Connectivity	ImagePro(Media Cybernetics)
Nearest Neighbor Analysis	15-500	Binary	None	Image J (NIH)
Cell Size, "Parent"	10- 300	(15 - 165)	8 Pixel Connectivity	ImagePro(Media Cybernetics)
Cell Soma, "Child"	20- 125	(25 - 125)	100%-filled holes	ImagePro(Media Cybernetics)
Sholl Analysis	7-125	Binary	Manual deletion of noise	Image J (NIH)

Supplementary Table 1. A review of the measure contraints used in the applications to estimate cell density and morphological changes.

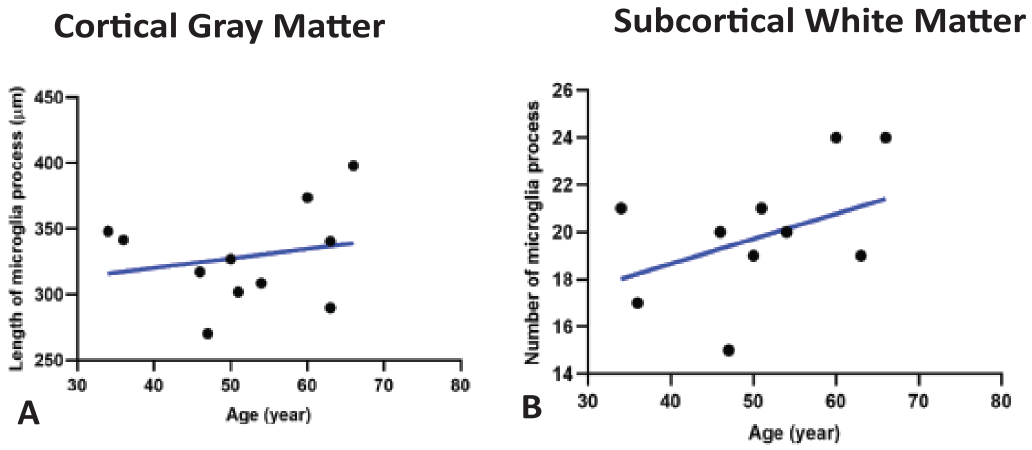

Supplementary Figure 2. The correlation between the ramification of microglia and age was not identified in control cases in either the cortical gray matter(A) or the subcortical white matter (B) of BA 9

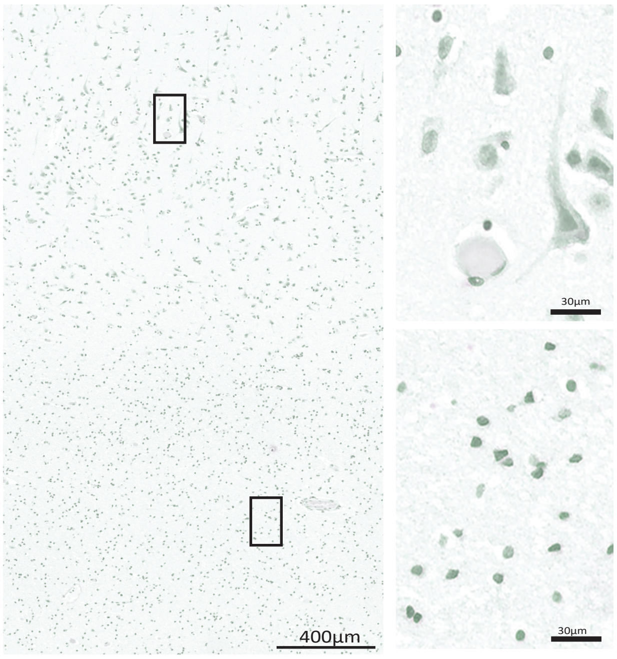

Supplementary Figure 3. A photomicrograph of a negative control from the BA 22 region (A) with higher magnification examples seen from the cortical gray matter (B) and the subcortical white matter region (C).
